# Traumatic brain injury is unlikely precipitating Leigh syndrome due to the GJB2 mutation c.35delG

**Published:** 2017-06-30

**Authors:** Josef Finsterer, Sinda Zarrouk-Mahjoub

**Affiliations:** 1 Krankenanstalt Rudolfstiftung, Vienna, Austria.; 2 University of Tunis El Manar and Genomics Platform, Pasteur Institute of Tunis, Tunis, Tunisia.

**Letter to the Editor**

With interest we read the article by Ashrafi et al. about a 14-year-old female who is regarded to have developed Leigh syndrome (LS) after traumatic brain injury (TBI) (1). We have the following comments and concerns:

We do not agree with the notion that traumatic brain injury was the precipitating factor for LS. The patient had a history of hypoacusis, which is a typical clinical manifestation of a mitochondrial disorder (MID). Hypoacusis obviously had developed long before the TBI. Additionally, the patient was diagnosed with neuropathy of the peripheral nerves two months after TBI. It is rather unlikely that neuropathy was triggered by TBI and more likely it was already present before the trauma. Thus, the initial manifestations of LS in the presented patient were most likely hypoacusis followed by neuropathy and TBI only might have triggered the seizure but not the MID. Why was the patient put on phenytoin, which is well-known to be mitochondrion-toxic (2)? Phenytoin may worsen epilepsy and MID in general and it is conceivable that in fact phenytoin was responsible for worsening of the phenotype and not the TBI. In a 16-year-old female with MELAS syndrome due to the mutation m.3243A>G, phenytoin caused intestinal pseudo-obstruction one month after intravenous phenytoin for status epilepticus (3). In a patient with Kearns-Sayre syndrome phenytoin decreased cerebrospinal fluid (CSF) folate levels (4). In rat hepatocytes, phenytoin increased reactive oxygen species (ROS) formation, decreased intracellular reduced glutathione, increased intracellular oxidised glutathione, and enhanced lipid peroxidation and mitochondrial damage (5). In a hepatic microsomal system, phenytoin decreased state-3 respiration, ATP synthesis, and the mitochondrial membrane potential. In this model, phenytoin increased state-4 respiration, impaired Ca++-uptake and release, and inhibited Ca++-induced swelling (6). It would be interesting to know how the *GJB2* mutation was detected. Was whole exome or panel sequencing carried out? Did the authors choose a gene by gene sequencing approach? Did the parents undergo genetic investigations? Did each of them carry the mutation in the heterozygous state? Did either of the parents manifest clinically? We should be informed about the exact sequence of the TBI. Is it conceivable that the fall was already due to a seizure or syncope? Was the patient unconscious after the TBI? The authors mention that basal ganglia lesions were “negative” on DWI (1). However, basal ganglia appear hyperintense on DWI in figure 2. What was the cause of respiratory insufficiency at the second admission? Was it attributable to the cerebral lesions, to myopathy affecting the respiratory muscles, to lactic acidosis, or to a pulmonary infection? Simultaneous application of a bunch of drugs is not advisable since it cannot be differentiated which of them is effective in case the administration is followed by a beneficial response. 

Overall, the report is not convincing with regard to TBI as the precipitating factor of LS. Most likely, worsening of LS was a random event or triggered by phenytoin. It is also conceivable that stress from TBI triggered the production of ROS, which were mitochondrion-toxic to such a degree that a previously subclinical condition became gradually symptomatic.
